# *Corni Fructus:* a review of chemical constituents and pharmacological activities

**DOI:** 10.1186/s13020-018-0191-z

**Published:** 2018-06-25

**Authors:** Yu Dong, Zhe-Ling Feng, Hu-Biao Chen, Fu-Sheng Wang, Jia-Hong Lu

**Affiliations:** 1 State Key Laboratory of Quality Research in Chinese Medicine, Institute of Chinese Medical Sciences, University of Macau, Room 7015, N22, Avenida da Universidade, Taipa, Macau SAR People’s Republic of China; 2 School of Chinese Medicine, Hong Kong Baptist University, Hong Kong, SAR People’s Republic of China; 3 Ulcerous Vascular Surgical Department, Beijing Traditional Chinese Medicine Hospital Affiliated to Capital Medical University, Beijing, People’s Republic of China

**Keywords:** *Cornus officinalis* Sieb. et Zucc., *Corni Fructus*, Shan Zhu Yu, Phytochemistry, Pharmacological activity

## Abstract

*Cornus officinalis* Sieb. et Zucc. is part of the genus *Cornus* of the family *Cornaceae*. Ripening and dry fruits (*Corni Fructus*) are recognized as an essential herb medicine in the traditional Chinese medicine (TCM) and have been widely used for over 2000 years. This review provides a comprehensive summary of *Corni Fructus* (CF), including the botany, phytochemistry, traditional use, and current pharmacological activities. According to the basic theory of TCM, CF usually participates in various Chinese medicinal formulae to exert the essential roles in replenishing liver and kidney, arresting seminal emission and sweat. Based on modern pharmacological studies, about 90 compounds have been isolated and identified from CF. In vivo and in vitro experimental studies indicate that CF exhibits extensive pharmacological activities including hypoglycemic, antioxidant, anti-inflammatory, anticancer, neuroprotective, hepatoprotective, and nephroprotective activities. However, only about 18% of chemical constituents in CF were tested. It means the potential pharmacological activities and clinical values of CF need to be further investigated.

## Background

*Cornus officinalis* Sieb. et Zucc., commonly known as Shan Zhu Yu/山茱萸 (in Chinese), Asiatic Dogwood, and Japanese Cornel Dogwood, is a deciduous shrub or dungarunga in the genus *Cornus* (family *Cornaceae*). It is a heliophilous plant that grows in the warm-temperate zone. The most suitable growth temperature is between 20 and 30 °C, it also has a specific cold resistance that can temporarily grow in − 18 °C low-temperature zone. *Cornus officinalis* Sieb. et Zucc. can be found in Anhui, Gansu, Jiangsu, Jiangxi, Shandong, Shanxi in China, Korea, and Japan. It usually grows in 400–1500 m high mountain slope, forest or forest edge. Ripening fruits are picked during September and October and dried in the air for medical uses [1, 2].

**Fig. 1:**
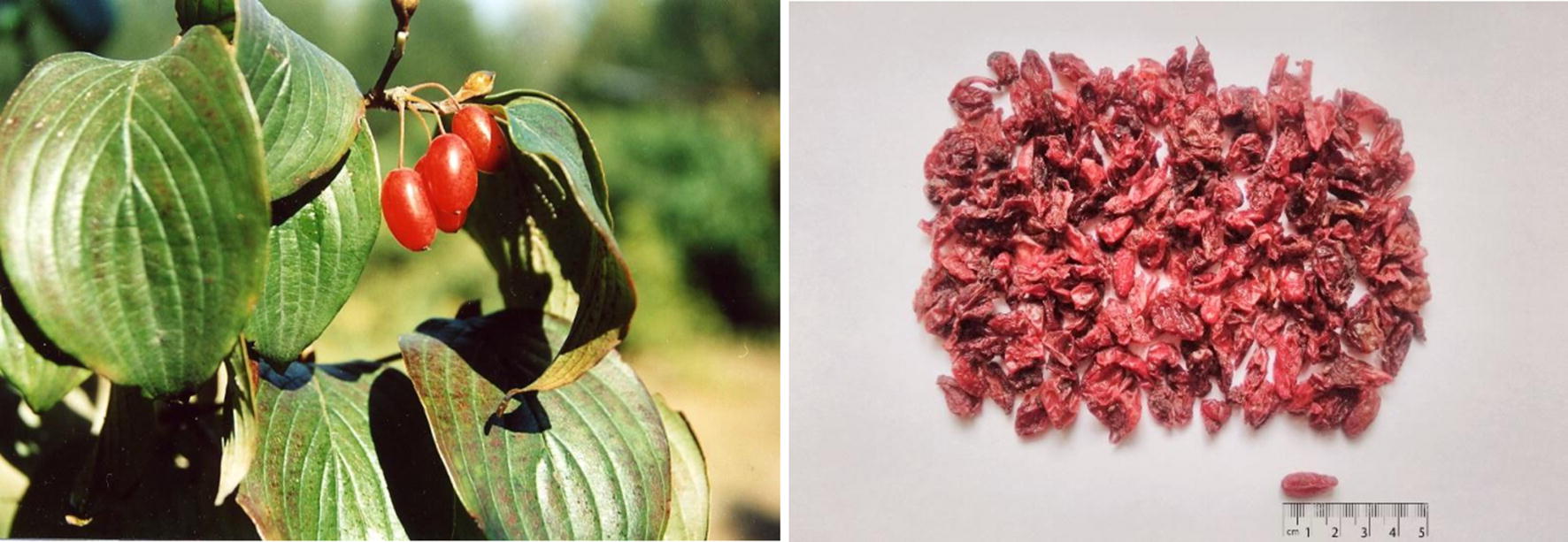
*Corni Fructus*: **a** crude fruits, **b** processed fruits

About 2200 years ago, *Cornus officinalis* Sieb. et Zucc. fructus (usually known as *Corni Fructus*) was first recorded in *Shen Nong’s Materia Medica* (Fig. 1). According to the basic theory of TCM, CF is characterized as replenishing liver and kidney, arresting seminal emission and sweat for its sour, astringent, and tepid properties [1]. It is used to treat four series of clinical symptoms. The first part of symptoms contains vertigo, tinnitus, weakness of the waist and knees which are caused by liver and kidney deficiency. CF is usually combined with *Radix Rehmanniae Praeparata*, *Dioscoreae Rhizoma*, *Alismatis Rhizoma*, *Moutan Cortex*, *Poria* to make Liuwei Dihuang Wan (六味地黄丸) replenish liver and kidney Yin [3]. For patients with kidney Yang deficiency, CF helps *Cinnamomi Cortex*, *Aconiti Lateralis Radix Praeparata* to reinforce Yang from Yin, e.g., Jingui Shenqi Wan (金匮肾气丸) [4]. The second part of symptoms contains spermatorrhoea and polydipsia. For patients with kidney deficiency, CF is frequently used with *Radix Rehmanniae Praeparata*, *Dioscoreae Rhizoma*, *Cervi Cornu Pantotrichum*, *Psoraleae Fructus*. For patients with dysfunction of the urinary bladder, CF is often applied with *Mantidis Oötheca*, *Rubi Fructus*, *Rosae Laevigatae Fructus*. The third part of symptom contains hypermenorrhea. CF is usually combined with *Radix Rehmanniae Praeparata*, *Angelica Sinensis*, *Radix Paeoniae Alba* to make Guchong Tang (固冲汤) preserve Primordial Qi and stop Blood [5]. The fourth part of symptoms contains profuse cold sweating, pale complexion, cold limbs, and a feeble pulse. For patients with the Yang depletion syndrome, *Ginseng Radix et Rhizoma*, *Aconiti Lateralis Radix Praeparata*, and CF are applied in Laifu Tang (来复汤) to restore Yang from collapse. Medical practices indicate that CF can be combined with either Yin-tonifying or Yang-invigorating herbs to act as the sovereign drug or adjuvant drug in Chinese medicinal formulae and treat different types of TCM syndromes. Besides, CF is primarily made into the honey bolus to treat chronic diseases while is usually made into the decoction to treat acute conditions.

## Chemical constituents

**Fig. 2:**
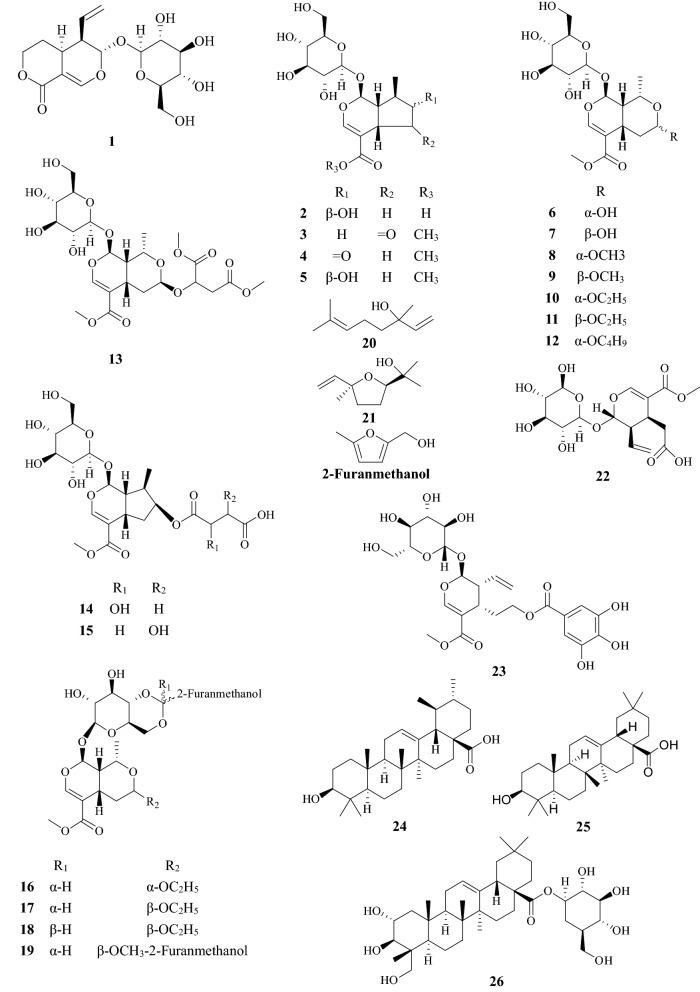
Structures of chemical constituents from *Corni Fructus*

**Fig. 3:**
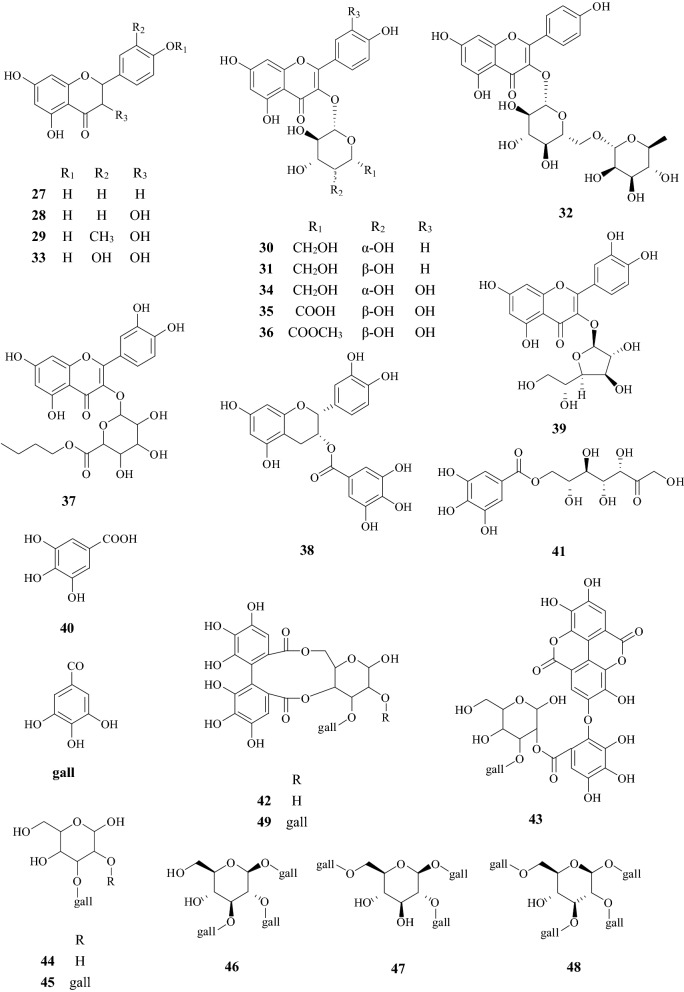
Structures of chemical constituents from *Corni Fructus*

**Fig. 4:**
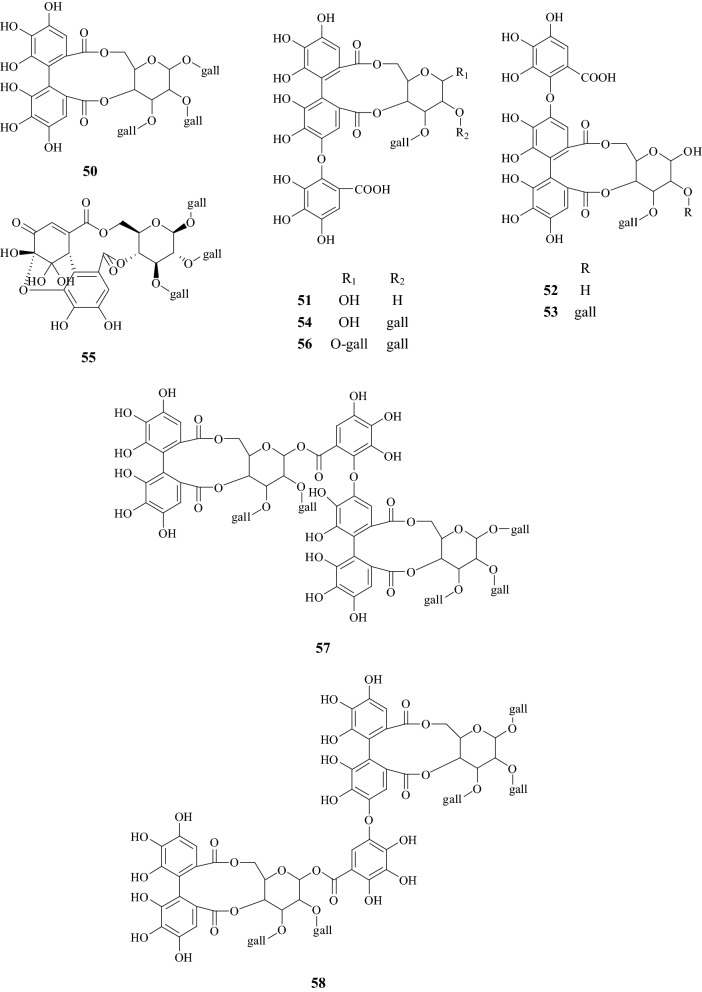
Structures of chemical constituents from *Corni Fructus*

**Fig. 5:**
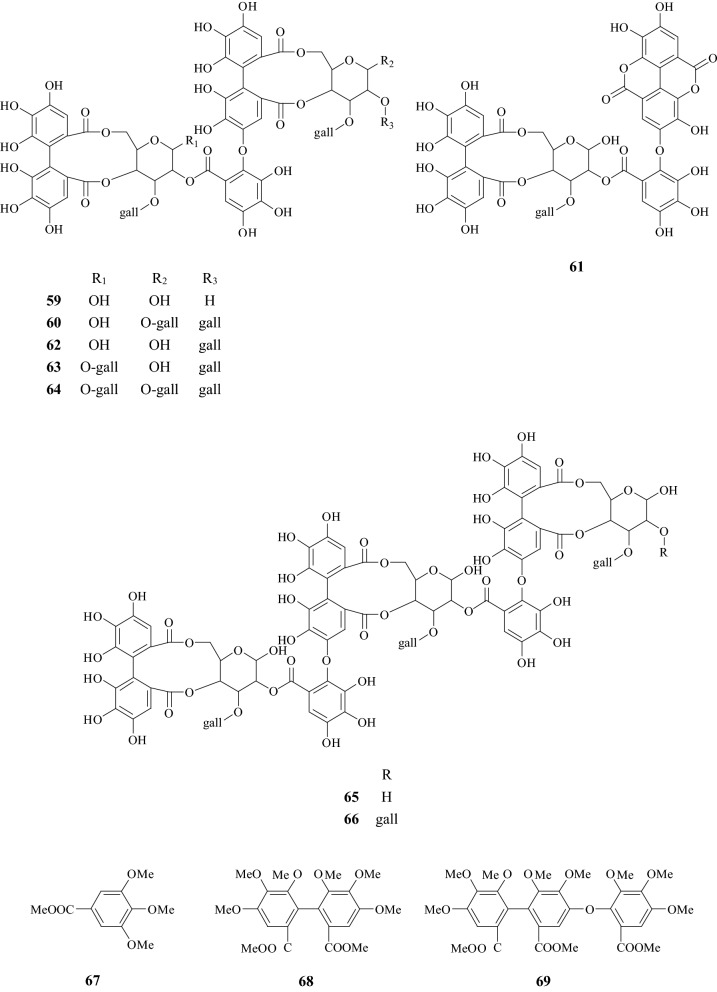
Structures of chemical constituents from *Corni Fructus*

About 90 compounds have been isolated and identified from CF, including terpenoids, flavonoids, tannins, polysaccharides, phenylpropanoids, sterols, carboxylic acids, furans, and mineral substances. Chemical constituents are listed in Table 1. Among them, iridoids, tannins, and flavonoids are the major components. Their chemical structures are shown in Figs. 2, 3, 4 and 5.

**Table 1:** Chemical constituents identified from CF

No.	Chemical class	Compound name	Chemical formula	Exact mass	References
	Terpenoids				
1	Iridoids	Sweroside	C_16_H_22_O_9_	358.1264	[73]
2		Loganic acid	C_16_H_24_O_10_	376.1369	[19]
3		Cornin	C_17_H_24_O_10_	388.1369	[73]
4		7-Dehydrologanin	C_17_H_24_O_10_	388.1369	[74]
5		Loganin	C_17_H_26_O_10_	390.1526	[74]
6		7-α-Morroniside	C_17_H_26_O_11_	406.1475	[75]
7		7-β-Morroniside	C_17_H_26_O_11_	406.1475	[75]
8		7-α-*O*-Methyl-morroniside	C_18_H_28_O_11_	420.1632	[75]
9		7-β-*O*-Methyl-morroniside	C_18_H_28_O_11_	420.1632	[75]
10		7-α-*O*-Ethyl-morroniside	C_18_H_29_O_11_	421.1710	[75]
11		7-β-*O*-Ethyl-morroniside	C_18_H_29_O_11_	421.1710	[75]
12		7-α-*O*-Butyl-morroniside	C_21_H_34_O_11_	462.2101	[43]
13		7-β-*O*-Dimethyl-butanedioate morroniside	C_23_H_34_O_15_	550.1898	[76]
14		Logmalicids A	C_21_H_29_O_14_	505.1557	[24]
15		Logmalicids B	C_21_H_29_O_14_	505.1557	[24]
16		Cornusfuroside A	C_25_H_34_O_13_	542.1999	[77]
17		Cornusfuroside B	C_25_H_34_O_13_	542.1999	[77]
18		Cornusfuroside C	C_25_H_34_O_13_	542.1999	[77]
19		Cornusfuroside D	C_29_H_34_O_15_	622.1898	[77]
20	Secoiridoids	Linalool	C_10_H_18_O	154.1358	[78]
21		Linalool oxide	C_10_H_18_O_2_	170.1307	[79]
22		Secoxyloganin	C_17_H_24_O_11_	404.1319	[24]
23		Cornuside	C_24_H_30_O_14_	542.1636	[74]
24	Triterpenoids	Ursolic acid	C_30_H_48_O_3_	456.3603	[74]
25		Oleanolic acid	C_30_H_48_O_3_	456.3603	[15]
26		Arjunglucoside II	C_36_H_58_O_10_	650.4030	[74]
	Flavonoids				
27		Naringenin	C_15_H_12_O_5_	272.0685	[74]
28		Kaempferol	C_15_H_10_O_6_	286.0477	[79]
29		Kaempferide	C_16_H_12_O_6_	300.0634	[80]
30		Kaempferol-3-*O*-β-d-galactopyranoside	C_21_H_20_O_11_	448.1006	[24]
31		Kaempferol-3-*O*-β-d-glucoside	C_21_H_20_O_11_	448.1006	[74]
32		Kaempferol-3-*O*-β-d-rutinoside	C_27_H_30_O_15_	594.1585	[24]
33		Quercetin	C_15_H_10_O_7_	302.0427	[79]
34		Quercetin-3-*O*-β-d-galactopyranoside	C_21_H_20_O_12_	464.0955	[24]
35		Quercetin-3-*O*-β-d-glucuronide	C_21_H_18_O_13_	478.0747	[24]
36		Quercetin-3-*O*-β-d-glucuronide methyl ester	C_22_H_20_O_13_	492.0904	[24]
37		Quercetin-3-*O*-β-d-(6-n-butyl glucuronide)	C_25_H_25_O_13_	533.1295	[15]
38		(−)-Epicatechin-3-*O*-gallate	C_22_H_18_O_10_	442.0900	[15]
39		Isoquercitrin	C_21_H_20_O_12_	464.0955	[80]
	Tannins				
40		Gallic acid	C_7_H_6_O_5_	170.0215	[74]
41		7-*O*-Galloyl-d-sedoheptulose	C_14_H_18_O_11_	362.0849	[19]
42		Gemin D	C_27_H_22_O_18_	634.0806	[6, 7]
43		Oenothein C	C_34_H_24_O_22_	784.0759	[6, 7]
44		3-*O*-Galloyl-d-glucose	C_13_H_16_O_10_	332.0743	[6, 7]
45		2,3-Di-*O*-galloyl-d-glucose	C_20_H_20_O_14_	484.0853	[6, 7]
46		1,2,3-Tri-*O*-galloyl-β-d-glucose	C_27_H_24_O_18_	636.0963	[6, 7]
47		1,2,6-Tri-*O*-galloyl-β-d-glucose	C_27_H_24_O_18_	636.0963	[6, 7]
48		1,2,3,6-Tetra-*O*-galloyl-β-d-glucose	C_34_H_28_O_22_	788.1072	[6, 7]
49		Tellimagrandin I	C_34_H_26_O_22_	786.0916	[6, 7]
50		Tellimagrandin II	C_41_H_30_O_26_	938.1025	[6, 7]
51		Isocoriariin F	C_34_H_26_O_23_	802.0865	[6, 7]
52		Coriariin F	C_34_H_26_O_23_	802.0865	[6, 7]
53		Rugosin B	C_41_H_30_O_27_	954.0974	[6, 7]
54		Isorugosin B	C_41_H_30_O_27_	954.0974	[6, 7]
55		Isoterchebin	C_41_H_30_O_27_	954.0974	[6, 7]
56		Isorugosin A	C_48_H_34_O_31_	1106.1084	[6, 7]
57		Rugosin D	C_82_H_58_O_52_	1874.1894	[6, 7]
58		Isorugosin D	C_82_H_58_O_52_	1874.1894	[6, 7]
59		Camptothin A	C_61_H_46_O_40_	1418.1565	[6, 7]
60		Camptothin B	C_75_H_54_O_48_	1722.1785	[6, 7]
61		Cornusiin B	C_48_H_30_O_30_	1086.0822	[6, 7]
62		Cornusiin A	C_68_H_50_O_44_	1570.1675	[6, 7]
63		Cornusiin D	C_75_H_54_O_48_	1722.1785	[6, 7]
64		Cornusiin E	C_82_H_58_O_52_	1874.1894	[6, 7]
65		Cornusiin F	C_95_H_70_O_62_	2202.2325	[6, 7]
66		Cornusiin C	C_102_H_74_O_66_	2354.2434	[6, 7]
67		Methyl tri-*O-*methylgallate	C_11_H_14_O_5_	226.0841	[6, 7]
68		Dimethyl hexamethoxydiphenate	C_22_H_26_O_10_	450.1526	[6, 7]
69		Trimethyl-octa-*O*-methylvaloneate	C_32_H_36_O_15_	660.2054	[6, 7]
	Polysaccharides				
70		Co-4			[8]
71		COP-1			[9]
72		COP-2			[9]
73		COP-3			[9]
74		COP-4			[9]
75		FCAP1			[81]
76		FCP5-A			[8]
77		PFCA-III			[8]
78		PFCC-I			[8]
79		SZYP-2			[8]
	Other compounds				
80	Phenylpropanoids	*p*-Hydroxycinnamic acid	C_9_H_8_O_3_	164.0473	[74]
81		Caffeic acid	C_9_H_8_O_4_	180.0423	[15]
82		Caftaric acid monomethyl ester	C_14_H_14_O_9_	326.2556	[15]
83		Caffeoyltartaric acid dimethyl ester	C_15_H_16_O_9_	340.0794	[76]
84	Sterols	β-Sitosterol	C_29_H_50_O	414.7067	[15]
85		Daucosterol-6′-malate	C_39_H_64_O_10_	692.4499	[80]
86	Carboxylic acids	Succinic acid	C_4_H_6_O_4_	118.0266	[85]
87		Malic acid	C_4_H_6_O_5_	134.0215	[85]
88		Methylmalic acid	C_5_H_8_O_5_	148.0372	[74]
89		Citric acid	C_6_H_8_O_7_	192.0270	[85]
90		Butoxysuccinic acid	C_8_H_14_O_5_	190.1938	[15]
91	Furans	5-Hydroxymethylfurfural	C_6_H_6_O_3_	126.0317	[73]
92		Dimethyltetrahydrofuran cis-2,5-dicarboxylate	C_8_H_12_O_5_	188.0685	[79]
93	Mineral substances	Ca, Fe, K, Mg, Mn, Zn			[82]

### Terpenoids (**1**–**26**) and flavonoids (**27**–**39**)

Most terpenoids and flavonoids in CF shared two similar isolation processes. Firstly, CF was percolated with ethanol to acquire the solvent which was then evaporated under reduced pressure. The resulting extract was suspended in water and then partitioned with ethyl acetate for several times. Finally, the extract was subjected to column chromatography over silica gel to yield compounds. Secondly, CF was grounded into powder and then subjected to supercritical carbon dioxide to yield extract. The resulting extract was subjected to GC–MS to identify the chemical components. So far, 26 terpenoids and 13 flavonoids have been isolated and identified from CF. Among terpenoids, the pharmacological activities of sweroside (**1**), loganin (**5**), cornuside (**23**), ursolic acid (**24**), and oleanolic acid (**25**) have been further assayed, and a wide range of pharmacological activities has been revealed. Furthermore, two types of flavonoids namely kaempferol (**28**), quercetin (**33**), and their derivatives are the essential flavonoids.

### Tannins (**40**–**69**)

During the isolation process, CF was firstly homogenized in acetone and then filtered to acquire an aqueous solution which was sequentially extracted with diethyl ether and ethyl acetate. The extract was subjected to column chromatography to give compounds. Finally, the chemical structure and molecular weight were determined using nuclear magnetic resonance (NMR) spectroscopy. To date, 30 tannic acids have been isolated from CF. Tsutomu HATANO identified 28 of them. Many tannic acids in this Chinese herb have the large molecular weight, e.g., the molecular weight of Cornusiins A–F and Camptothins A–B are even larger than 1000 Da [6, 7], because dimers and trimers exist in these types of tannic acids.

### Polysaccharides (**70**–**79**)

Wu and Yin identified most polysaccharides in CF [8, 9]. In their isolation process, hot water or petroleum ether was initially used for combining with assistant ultrasonic and microwave to break the cell wall to isolate polysaccharides. Further separation and purification were achieved by the combination of several techniques, e.g., fractional precipitation, ethanol precipitation, ion-exchange chromatography and affinity chromatography. Finally, infrared spectroscopy analysis and morphological analysis were used to determine the physiochemical and structural features of the polysaccharide.

### Other compounds (**80**–**93**)

Four phenylpropanoids, two sterols, five carboxylic acids, two furans, and several mineral substances have also been determined. Among them, 5-hydroxymethylfurfural exhibits diverse biological activities. Besides, Chen, Li, and Wen identified 32, 16, and 48 volatile compounds by GC–MS, respectively [10–12].

## Pharmacological activities

Although just a few chemical constituents from CF are assayed for their biological activities, these components displayed diverse pharmacological activities. Detailed biological activities are summarized in Table 2.

**Table 2:** Summary of pharmacological activities of CF

Extracts or compounds	Disease models	Specific effects	References
Hypoglycemic activity			
Oleanolic acid	Fasting rat	Decrease plasma glucose levels. Regulate ACh release from nerve terminals to activate muscarinic M3 receptors in the pancreatic cells and increase C-peptide and insulin release	[83]
Iridoid glycosides	STZ-induced rat as DM model	Show α-glucosidase inhibition activity in vitro and decrease serum glucose levels in vivo	[16]
Loganin Morroniside Ursolic acid	STZ-induced mice as DM model, HepG2 cell lines	Show α-glucosidase inhibition activity in vitro. Decrease fasting blood glucose and alleviate weight loss, polydipsia, and polyphagia. Increase SOD activity and ROS scavenging activity. Attenuate aldose reductase activity and decrease MDA plasma level and renal somatic indices in mice	[14]
Butyl morroniside (−)-Epicatechin-3-*O*-gallate Caftaric acid monomethyl ester	High glucose-induced BRIN-BD11 and H4IIE cell lines as in vitro DM model	Increase glucose uptake efficiency. Reduce PEPCK mRNA level and NO production. Inhibit pancreatic β-cell death	[15]
Aqueous extract	STZ-induced rat as diabetic organs injury model	Decrease levels of glucose and TC in serum, and α-SMA expression in kidney. Improve the pathohistological injury of pancreas, kidney, lung, and liver	[13]
Aqueous extract	Normal rat	Show α-glucosidase inhibition activity in vitro, and exhibit hypoglycemic effect via oral sucrose tolerance test in vivo	[17]
Aqueous extract	Dexamethasone and 8-bromo-cAMP-induced BRIN-BD11 and H4IIE cell lines as in vitro DM model	Increase insulin release. Decrease PEPCK mRNA level	[18]
Nephroprotective activity			
Loganin	STZ-induced rat and high glucose-induced HK-2 as in vivo and in vitro diabetic nephropathy model	Improve renal function. Decrease CTGF level in kidney and serum via ERK signaling pathway	[23]
Morroniside Loganin 7-*O*-Galloyl-d-sedoheptulose	Db/db mice as obesity-associated type 2 diabetic nephropathy model	Suppress formation of AGEs and TBARS in the kidney. Reduce the production of SREBP-1&2, NF-κB p65, COX-2, and iNOS. Decrease GSH/GSSG ratio and levels of serum glucose, TC, and TG	[19]
7-*O*-Galloyl-d-sedoheptulose	STZ-induced rat as diabetic nephropathy model	Decrease serum creatinine, renal glucose, and urinary protein. Reduce the production of AGE, RAGE, HO-1, intracellular glycation, CML, GA-pyridine, and TBARS	[21]
Iridoid glycosides	STZ-induced rat as diabetic nephropathy model	Suppress over-deposition of fibronectin and laminin in the kidney. Reduce protein and mRNA levels of TGF-β1 in serum and glomeruli	[25]
Iridoid glycosides Triterpene acids	Db/db mice as obesity-associated type 2 diabetic nephropathy model	Improve the histological injury of kidney and pancreas. Ameliorate the structural alterations in mesangial cells and the podocytes in the renal cortex. Inhibit ECM accumulation in the kidney. Decrease 24 h urine protein and serum levels of urea nitrogen and creatinine. Increase insulin release, and decrease fasting blood glucose and levels of TC, TG, and GSP. Attenuate food consumption, water intake, and urine volume. Reduce the production of RAGE, NF-κB, SphK1, and TGF-β	[22]
CF extract	STZ-induced rat as diabetic nephropathy model	Inhibit AGE formation in the kidney. Attenuate hyperglycemia and proteinuria. Reduce the production of RAGE, NF-κB, TGF-β1, and CML	[20]
Ethanol extract	High glucose-induced mesangial cells as in vitro diabetic nephropathy model	Decrease the production of Col V, FN, and IL-6	[24]
Myocardial protection activity			
Morroniside	High glucose-induced rat as diabetic cardiomyopathy model	Inhibit myocardial cell apoptosis. Elevate Bcl-2 production and decrease expressions of Bax and caspase-3	[28]
Triterpene acids	STZ-induced rat as diabetic cardiomyopathy model	Inhibit the ventricular remodeling and regulate the systolic and diastolic function of the left ventricle. Increase insulin release and reduce serum glucose levels. Enhance GSX and SOD activity. Increase the production of calstabin 2, PLB, and SERCA2a. Decrease protein and mRNA levels of ECE, iNOS, MDA, ET-1, and propreET-1	[26]
Testis-protective activity			
Iridoid glycosides	STZ-induced rat as diabetic testicular damage model	Improve the pathohistological injury of testes and pancreas. Increase serum insulin release and decrease blood glucose levels. Alleviate weight loss, polydipsia, polyphagia, and polyuria. Increase CAT and SOD activity. Reduce the production of AGEs, RAGE, ROS, MDA, and p-p38 MAPK. Down-regulate Bax/Bcl-2 ratio and spermatogenic cell apoptosis	[27]
Antioxidant activity			
Morroniside	Hydrogen peroxide-induced SH-SY5Y cell line as in vitro neurodegenerative disorder model	Suppress intracellular accumulation of Ca^2+^. Increase SOD activity and reduce the loss of MMP. Inhibit cytotoxicity	[29]
Morroniside	High ambient glucose-induced endothelial cell injury model	Attenuate cellular morphological damage. Repair cell cycle progression and improve cell viability	[35]
Ursolic acid	Hydrogen peroxide-induced HEI-OC1 cell line as in vitro inner ear diseases model	Increase antioxidant enzymes expressions, e.g., CAT and GPX. Suppress lipid peroxidation	[32]
5-Hydroxymethylfurfural	High glucose-induced HUVECs as in vitro oxidative stress model	Decrease levels of ROS, IL-8, JNK1, and JNK2/3. Increase P-Akt production	[34]
Total saponins	STZ-induced rat as a diabetic oxidative stress model	Regulate NO release and endothelium-dependent relaxation on the mesenteric artery. Reduce blood glucose levels	[30]
Aqueous extract	Hypoxanthine and xanthine oxidase-induced bovine PAECs as in vitro oxidative stress model	Regulate GSH redox cycle. Increase the intracellular GSH production and the activity of GSH peroxidase and GSH disulfide reductase. Reduce the intracellular level of GSH disulfide. Increase CAT and SOD activity and inhibit the production of hydrogen peroxide and superoxide anion	[31]
Ethanol extract	LPS-induced RAW 264.7 macrophage cells as in vitro oxidative stress model	Attenuate xanthine oxidase activity and ROS production. Induce the production of antioxidant enzymes, e.g., CAT, GSX, Cu/Zn-SOD, and Mn-SOD	[33]
Anti-inflammatory activity			
Cornuside	TNF-α-induced HUVECs as in vitro inflammation model	Decrease the production of ICAM-1, VCAM-1, MCP-1, and NF-κB. Inhibit NF-κB p65 translocation	[36]
Cornuside	LPS-induced RAW 264.7 macrophage cells as in vitro inflammation model	Decrease the production of COX-2, iNOS, PGE_2_, NO, IL-1β, IL-6, and TNF-α. Suppress the translocation of NF-κB p65, the phosphorylation and degradation of IκB-α, and the phosphorylation of ERK1/2, JNK1/2, and p38	[38]
Aqueous extract	LPS-induced RAW 264.7 macrophage cells as in vitro inflammation model	Decrease protein and mRNA levels of COX-2 and iNOS. Reduce PGE_2_ and NO production	[37]
Anticancer activity			
Aqueous extract	HSC-2, HSC-3, HSC-4, Ca9-22, NA cell lines as in vitro oral squamous cell carcinoma model	Produce broad radical peak under alkaline condition and increase the cytotoxicity and superoxide anion scavenging activity of vitamin C	[39]
Aqueous extract	E2-induced MCF-7 cell line as in vitro ER^+^ human mammary carcinoma model	Inhibit cell line anchorage-independent growth and reduce the mitogenically inert metabolite E3 formation	[40]
Aqueous extract	Parental ER^+^ MCF-7 cell line as in vitro human mammary carcinoma model	Suppress cell line anchorage-independent growth and induce G1 or G2/M arrest and apoptosis. Increase anti-proliferative E2 metabolites production	[41]
Aqueous extract	HepG2, SKHep1 and PLC/PRF/5 cell lines as in vitro hepatocellular carcinoma model	Inhibit cell proliferation. Exhibit free radicals scavenging activity and suppress lipid peroxidation and xanthine oxidase production	[42]
Neuroprotective activity			
Cornuside 1,2,3-Tri-*O*-galloyl-β-d-glucose 1,2,3,6-Tetra-*O*-galloyl-β-d-glucose Tellimagrandin I Tellimagrandin II Isoterchebin	In vitro enzyme activities assay	Exhibit synergetic inhibitory activities against BACE1 and ChE	[45]
Morroniside	MCAO-induced rat as focal cerebral ischemia model	Decrease the infarction volume and improve neurological function. Increase GSH expression and SOD activity. Decrease the production and activity of MDA and caspase-3 in ischemic cortex tissues	[47]
5-Hydroxymethylfurfural	Hydrogen peroxide-induced rat hippocampal neurons as in vitro neurodegenerative disorder model	Enhance Bcl-2 production and suppress expressions of Bax, caspase-3, and p53	[84]
Iridoid glycosides	MCAO-induced rat as focal cerebral ischemia model	Improve neurological function. Increase the number of BrdU-positive cells and nestin-positive cells in the subventricular zone, and the number of new mature neurons and blood vessels in the striatum. Increase protein and mRNA levels of VEGF and Flk-1	[46]
Iridoid glycosides	Fimbria-fornix transected rat as cerebral ischemia model	Decrease neuron loss in the hippocampus and improve memory deficits. Increase the production of BDNF, NGF, Bcl-2, SYP, Trk A, and GAP-43, and decrease the production of Bax and Cyt c	[49]
7*R*-*O*-Methyl-morroniside 7*S*-*O*-Methyl-morroniside 7-*O*-Butyl-morroniside Loganin Morroniside	Glutamate-induced HT22 cell lines as in vitro hippocampal cell injury	Improve cell viability	[43]
Iridoid glycosides	*Mycobacterium tuberculosis* and guinea-pig myelin basic protein-induced experimental autoimmune encephalomyelitis rat as multiple sclerosis model	Increase the number of mature oligodendrocytes and reduce the number of oligodendrocyte progenitor cells. Inhibit the process of T cell entry to the central nervous system and attenuate microglia activation. Increase BDNF expression and decrease phosphorylation of JAK/STAT1/3 and inflammatory cytokines production, e.g., IL-1β, IFN-γ, TNF-α	[48]
Iridoid glycosides	*Mycobacterium tuberculosis* and myelin oligodendrocyte glycoprotein-induced experimental autoimmune encephalomyelitis mouse as multiple sclerosis model	Decrease BDNF and NGF loss in the spinal cord	[50]
Aqueous extract	PC 12 cell lines	Increase cell neurite outgrowth. Inhibit extracellular Ca^2+^ influx, and protein and mRNA levels of STIM1	[44]
Hepatoprotective activity			
5-Hydroxymethylfurfural	Hydrogen peroxide-induced L02 cell lines as in vitro hepatitis model	Promote S phase into G2/M phase and recover cell cycle to normal. Reduce NO production and caspase-4 activity and inhibit hepatocyte apoptosis	[51]
5-Hydroxymethylfurfural	Hydrogen peroxide-induced L02 cell lines as in vitro hepatitis model	Improve hepatocyte morphology and reduce caspase-3&9 expressions	[52]
5-Hydroxymethylfurfural	d-Galactosamine/TNF-α-induced L02 cell lines as in vitro acute liver injury model	Inhibit hepatocyte apoptosis. Increase Bcl-2 production and decrease intracellular Ca^2+^ level and production of ATF4, Bax, CHOP, PERK, and p-eIF2α	[53]
7-*O*-Galloyl-d-sedoheptulose	Db/db mice as obesity-associated type 2 diabetic liver injury model	Improve hepatic histological damage and decrease serum levels of ALT, AST, and blood glucose. Attenuate water intake, food consumption, and body weight gain. Decrease the production of AP-1, NF-κB p65, IL-6, TNF-α, ICAM-1, MCP-1, AGEs, RAGE, GA-pyridine, pentosidine, CEL, CMA, CML, leptin, resistin, p-ERK1/2, and p-JNK	[54]
Ethanol extract	Acetaminophen-induced mice as liver injury model	Increase levels of CAT, HO-1, and SOD. Suppress lipid peroxidation	[55]
Improving osteoporosis activity			
Sweroside	Rat osteoblasts and human MG-63 cell lines	Stimulate the osteocalcin secretion. Increase cell proliferation and inhibit apoptotic cell death. Increase ALP activity	[56]
CF extract	RANKL-induced mice BMDM as in vitro osteoclast differentiation model	Suppress osteoclast differentiation. Reduce protein and mRNA levels of c-Fos, NFATc1, OSCAR, and TRAP. Inhibit phosphorylation of p-38 and c-JNK and degradation of I-κB	[57]
Promoting melanogenesis activity			
Methanol extract	Melan-a cell lines	Increase the production and activity of tyrosinase. Increase MITF-M mRNA level and TRP-1&2 production	[58]
Immunomodulatory activity			
Aqueous extract	C57BL/6 mice are transplanted with a skin graft from Balb/C donors	Prolong skin allograft survival. Reduce the number of graft-infiltrating T cells and inhibit their proliferation. Decrease intracellular IL-12 expression by intragraft DCs and IFN-γ expression by graft-infiltrating T cells. Reduce intragraft IL-12 mRNA level	[59]
Lung-protective activity			
Oleanolic acid Ursolic acid	Epidermal growth factor—and phorbol ester‐induced NCI‐H292 cell lines as in vitro airway diseases model	Decrease protein and mRNA levels of MUC5AC mucin	[60]
Aqueous extract	Ovalbumin-induced BALB/c mice as allergic asthma model	Inhibit eosinophil infiltration and ameliorate allergic airway inflammation and airway hyperresponsiveness. Decrease the production of IL-5&13 and OVA-specific IgE	[61]
Vasorelaxation activity			
Cornuside	Phenylephrine-contracted rat aorta and HUVEC	Dilate vascular smooth muscle in the rat and increase cGMP production in vitro	[62]
Antiviral activity			
Aqueous extract	CVA16 infected Vero cells as in vitro HFMD model	Inhibit CVA16 replication	[63]

### Hypoglycemic activity and diabetic target organs protective activity

Diabetes mellitus (DM) is a group of long-term and chronic metabolic disorders which are associated with high serum glucose levels. Compared with the no treatment diabetic animal model group, CF extract (at 300 mg kg^−1^ 2 day^−1^ and 400 mg kg^−1^ day^−1^ p.o.), loganin, morroniside, and ursolic acid (each at 200 mg kg^−1^ day^−1^ p.o.) for 4 weeks can significantly decrease fasting blood glucose and alleviate polyphagia, polydipsia, polyuria, and weight loss [13, 14]. In He’s study, metformin (at 200 mg kg^−1^ day^−1^ p.o.) demonstrated better effect [14]. Besides, loganin, morroniside, ursolic acid, and butyl morroniside (each at 100 μmol L^−1^) can protect the pancreatic β-cells from high glucose-induced excessive oxidative stress and apoptosis [14, 15], may further increase the insulin release. Compared with the insulin treatment, CF extract, (−)-epicatechin-3-*O*-gallate, and caftaric acid monomethyl ester (each at 50 μmol L^−1^) can also significantly inhibit α-glucosidase activity to slow down the elevation of serum glucose levels [14, 16, 17] and suppress the hepatic gluconeogenesis by decreasing the protein and mRNA levels of PEPCK in vitro [15, 18].

Also, CF extract, iridoid glycosides, and the single compound can decrease 24 h urine protein and serum levels of urea nitrogen and creatinine. To be specific, loganin, morroniside, and 7-*O*-galloyl-d-sedoheptulose (each at 20–100 mg kg^−1^ day^−1^ p.o.) for 10 days and 8 weeks can significantly inhibit both AGE/RAGE formation [19–22] and CTGF production [23] in db/db mice or STZ-induced diabetic nephropathy model. They can also significantly alleviate diabetic organ injury by decreasing the production of NF-κB and its downstream synthetases and cytokines [19–25], increasing antioxidant enzyme production [19, 26, 27], and suppressing apoptotic cell death [27, 28].

### Antioxidant activity

Long-term oxidative stress will generate excessive ROS to oxidize protein, lipids, DNA and then cause cell death, tissue damage, and organ dysfunction. Ideal antioxidant drugs are required to regulate the defense system and scavenge excessive ROS. Studies indicated that morroniside (at 1, 10, 100 μmol L^−1^) for 24 h and total saponins (at 60 and 120 mg kg^−1^ day^−1^ p.o.) for 4 weeks regulated Ca^2+^ and NO release [29, 30], the aqueous extract (at 0.25–2.0 mg mL^−1^) for 20 h modulated GSH redox cycle [31], the aqueous extract, the ethanol extract (at 0.01–0.1 mg mL^−1^), morroniside (at 0.05–2 µg mL^−1^), and ursolic acid (at 0.05–2 µg mL^−1^) for 24 h promoted antioxidant enzymes syntheses [31–33] to inhibit lipid peroxidation [29], 5-hydroxymethylfurfural (at 100–400 μmol L^−1^) for 3 days decreased ROS release [34], morroniside (at 100 μmol L^−1^) for 2 days recovered cell cycle to normal state [35]. Mentioned effects significantly together reduced the oxidative stress-induced damages compared with the no treatment group.

### Anti-inflammatory activity

Prolonged and incurable inflammation may cause many diseases, e.g., atherosclerosis, cancer, ulcerative colitis. In LPS and TNF-α-induced cell inflammation models, compared with the no treatment group, CF aqueous extract (at 0.2, 1, 5 mg mL^−1^) and cornuside (at 1, 10, 50 μmol L^−1^) for 24 h significantly inhibited NF-κB p65 translocation, down-regulated COX-2 and iNOS production, finally decreased PGE_2_ and NO levels to control excessive inflammatory responses [36–38].

### Anticancer activity

CF aqueous extract significantly enhanced both the cytotoxicity and superoxide anion scavenging activity of vitamin C at 0.5 and 36 µg mL^−1^, respectively. Together with CF aqueous extract, vitamin C further inhibited proliferation and induced apoptosis in several human oral squamous cell carcinoma cell lines. Compared with no treatment, the proliferation inhibition rate was at 1.3–71.0% [39]. Furthermore, the aqueous extract (at 1.0 mg mL^−1^) for 2 days significantly exhibited anti-ER^+^ human mammary carcinoma activity by inhibiting cell anchorage-independent growth, regulating the metabolism of E2 and E3 [40], and influencing cell cycle progression and cellular apoptosis [41]. Finally, the aqueous extract has been tested for its cancer inhibitory effect in several hepatocellular carcinomas and leukemic cell lines. The study indicated that the aqueous extract inhibited the tumor cell proliferation in a dose-dependent manner at 0.11–0.337 mg mL^−1^, exhibited oxygen free radicals scavenging activity (at 50 µg mL^−1^), attenuated xanthine oxidase production (at 2.62 mg mL^−1^) and lipid peroxidation (at 0.892 mg mL^−1^) [42]. In this study, CF aqueous extract exhibited the similar effects compared with 5-fluorouracil (at 0.5, 1, 5 µg mL^−1^).

### Neuroprotective activity

Many compounds in CF were further tested for the neuroprotective effects. 7*R*-*O*-Methyl-morroniside, 7*S*-*O*-methyl-morroniside, 7-*O*-butyl-morroniside, loganin, and morroniside (each at 10 and 50 μmol L^−1^) for 1 h significantly protected the neurons against glutamate-induced neurotoxicity up to about 78% compared with the no treatment group [43]. CF aqueous extract (at 60 µg mL^−1^) significantly inhibited the extracellular Ca^2+^ influx to increase cell neurite outgrowth [44]. Also, cornuside, isoterchebin, and tellimagrandin II (each at 25–100 μmol L^−1^) displayed anti-Alzheimer’s disease potential due to their synergetic inhibitory activities against BACE1 and ChE [45].

Cerebral ischemia, multiple sclerosis, and neurodegenerative disorder models are applied in animal experiments. Iridoid glycosides (at 60 and 180 mg kg^−1^ day^−1^ p.o.) for 1–4 weeks and morroniside (at 90 and 270 mg kg^−1^ day^−1^ p.o.) for 3 days significantly decreased the infarction volume, increased the number of new mature neurons and blood vessels, and improved nervous system function [46, 47]. Also, iridoid glycosides (at 50–180 mg kg^−1^ day^−1^ p.o.) for 3–4 weeks can significantly promote NGF and BDNF production [48–50], and repair the abnormal functions of microglia, oligodendrocyte, and T cell to maintain the central nervous system homeostasis [48].

### Hepatoprotective activity

In hepatitis cell models, 5-hydroxymethylfurfural (at 0.2–1 and 0.79 μmol L^−1^) for 24 h has been shown to protect hepatocytes from H_2_O_2_ induced-cytotoxicity by significantly decreasing NO and intracellular Ca^2+^ levels, inhibiting abnormal production of apoptosis-related proteins and recovering back to regular cell cycle [51–53]. In hepatitis animal models, 7-*O*-galloyl-d-sedoheptulose (at 20 and 100 mg kg^−1^ day^−1^ p.o.) for 6 weeks and CF ethanol extract (at 100–500 mg kg^−1^ day^−1^ p.o.) for 1 week significantly decreased the serum marker enzymes of hepatic damage, weakened the oxidative stress by promoting antioxidant enzymes production and inhibiting lipid peroxidation, finally improved hepatic histological injury [54, 55].

### Other pharmacological activities

In addition to the mentioned pharmacological activities, CF has also been reported to exert multiple bioactivities. Firstly, sweroside (at 7.5 µg mL^−1^) for 1 week significantly promoted the proliferation and differentiation of osteoblasts via the regulation of osteocalcin [56]. Also, CF extract (at 0–100 µg mL^−1^) for 4 days significantly inhibited osteoclast differentiation in a dose-dependent manner via the inhibition of the signaling cascades NF-κB/c-Fos/NFATc1 to improve osteoporosis [57]. Secondly, CF methanol extract (at 3.125–12.5 µg mL^−1^) for 3 days significantly up-regulated synthesis and activity of tyrosinase, raised TRP-1&2 translation associating with increasing transcription of MITF-M, finally promoted melanogenesis by 36.1% [58]. Thirdly, CF aqueous extract possesses immunomodulatory activity. In C57BL/6 mice that were transplanted with a skin graft from Balb/C donors, CF extract significantly prolonged skin allograft survival synergistically by suppressing Th1 response, promoting regulatory T cell generation, and enhancing its suppressive function [59]. Fourthly, CF shows lung-protective activity via two studies. In the cellular test, oleanolic acid (at 10 and 100 μmol L^−1^) and ursolic acid (at 100 μmol L^−1^) for 30 min’ pretreatment significantly down-regulated MUC5AC mucin whose excessive level would impair airway defenses to cause serious airway diseases [60]. In an animal experiment, CF aqueous extract (at 50 and 200 mg kg^−1^ 3 day^−1^ p.o.) for 5 weeks significantly decreased the production of inflammatory mediators and reduced eosinophil infiltration, finally attenuated allergic airway inflammation and airway hyperresponsiveness [61]. Fifthly, cornuside significantly dilated vascular smooth muscle in phenylephrine-contracted rat aorta via the up-regulation of cGMP level to show its vasorelaxation activity [62]. Finally, among in vitro screening of antiviral drugs for treating hand, foot, and mouth disease (HFMD) infection, CF aqueous extract (at 0.4 µg mL^−1^) for 2 days significantly inhibited CVA16 replication in cellular level [63].

## Conclusion

CF is recognized as a fundamental constituent part of tonifying Yin and Yang prescription because of its harmonious and complementary features according to the basic theory of TCM. It possesses the properties of sour and astringent. Firstly, sour and sweet herbs can be combined to nourish Yin, it can act as the sovereign and ministerial drug among *Radix Rehmanniae Praeparata*, *Dioscoreae Rhizoma*, *Lycii Fructus*, *Ligustri Lucidi Fructus*, *Schisandrae Chinensis Fructus*. Also, sour and astringent properties exhibit their function of astringing and storing. It also behaves as the sovereign and the ministerial drug that combines with *Euryales Semen*, *Sepiae Endoconcha*, *Mantidis Oötheca*, *Rubi Fructus*, *Paeoniae Radix Alba* to treat spermatorrhea, urorrhagia, metrorrhagia and metrostaxis, and excessive perspiration. Finally, CF can be as the adjuvant and conductant drug to alleviate warm and dry features of Yang-reinforcing drugs.

**Fig. 6:**
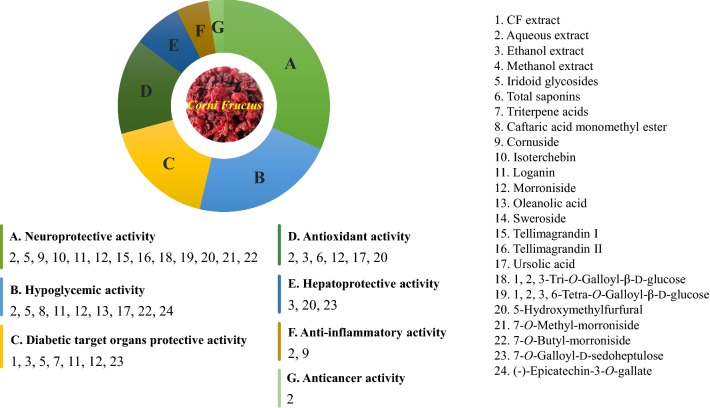
The various pharmacological activities of the extract and chemical compounds identified from *Corni Fructus*

Chemical constituents from terpenoids, flavonoids, tannins, and furans exhibited diverse biological activities, including hypoglycemic, neuroprotective, heart-protective, hepatoprotective, nephroprotective, testis-protective activities. Pharmacological activities are outlined in Fig. 6. In these studies, bioactive components from CF mainly alleviated the damage of target organs by antioxidant activity, anti-inflammatory activity, and anti-apoptosis activity, i.e., up-regulating the expressions and activities of antioxidant enzymes, down-regulating the levels of cytokines and chemokines, and modulating the abnormal expressions of apoptotic death associated proteins.

Hypoglycemic activity and alleviating diabetic target organs damage are critical pharmacological activities among the broad spectrum of pharmacological activities of CF. Morroniside, loganin, oleanolic acid, ursolic acid, and 7-*O*-galloyl-d-sedoheptulose exhibited the similar efficacy compared with the conventional oral hypoglycemic drugs (acarbose and metformin). In vivo studies, they reduced serum glucose levels and alleviated unusual symptoms caused by diabetes. In cellular assays, they protected pancreatic β cell from oxidative damage, increased insulin release, improved insulin resistance, displayed α-glucosidase inhibition activity, and suppressed liver gluconeogenesis. Also, compounds alleviated the high-glucose triggered target organs damage by attenuating oxidative stress, inflammation, and apoptosis, finally kept the essential function of target organs stable. CF has also been widely used to treat DM in clinical work. For example, Jingui Shenqi Wan and Liuwei Dihuang Wan are two classic Chinese medicinal formulae which contain CF. Clinical trials indicated that Jingui Shenqi Wan and Liuwei Dihuang Wan could decrease serum glucose levels, alleviate typical DM symptoms and repair target organs injury [64–67]. Diverse anti-diabetes and anti-diabetic complication pharmacological activities make CF a potential herb to become the complementary drug for treating DM.

Another significant biological activity is the neuroprotection. In cerebral ischemia rat model and neurodegenerative disorder cellular model, iridoid glycosides (e.g., morroniside) and 5-hydroxymethylfurfural increased the number of new mature neurons and blood vessels and exerted anti-oxidative stress, anti-inflammation, and anti-apoptosis properties. In cerebral ischemia rat model and multiple sclerosis rats and mice models, iridoid glycosides also enhanced the levels of brain-derived neurotrophic factor and nerve growth factor. Current studies showed that the pathogenic mechanisms of neurodegenerative diseases have the close relationship with autophagy deficiency and abnormal proteins aggregate clearance dysfunction [68, 69]. In addition to the anti-apoptotic activity, pharmacological activities of CF on the regulation of autophagy can be further explored. Furthermore, many classic Chinese medicinal formulae have been used to treat neurological disorders belonging to liver and kidney deficiency [70–72]. For example, Liuwei Dihuang Wan treats insomnia, Zuogui Wan (左归丸) treats epilepsy and vertigo, Dabu Yinjian (大补阴煎) treats a headache, Zuogui Wan and Dihuang Yinzi (地黄饮子) treats stroke, and Huanshao Dan (还少丹) treats dementia. CF plays a vital role in nourishing liver and kidney Yin in these Chinese medicinal formulae.

However, about 90 compounds have been isolated and identified from CF, only 18% compounds are further assayed for their pharmacological activities in vivo and in vitro. It indicates that pharmacological activities of the remaining 90% chemical components are still unknown yet. Moreover, current studies do not provide enough evidence to verify the drug binding sites of active ingredients of CF. For example, it is difficult to judge whether these active ingredients bind the G protein coupled receptor, ion channels, transmembrane receptor kinases, or nuclear receptors to work. Therefore, more systematic and detailed pharmacological studies on CF need to be fulfilled in the future.

## Abbreviations

ACh: acetyl choline
AGEs: advances glycation endproducts
ALP: alkaline phosphatase
ALT: alanine aminotransferase
AST: aspartate aminotransferase
ATF4: activating transcription factor 4
BACE1: b-site amyloid precursor protein cleaving enzyme 1
Bax: bcl-2-associated X
Bcl-2: B-cell lymphoma-2
BDNF: brain-derived neurotrophic factor
BMDM: bone marrow-derived macrophages
BrdU: bromodeoxyuridine
CAT: catalase
CEL: Ne-(carboxyethyl)lysine
ChE: cholinesterase
CHOP: C/EBP homologous protein
CMA: Ne-(carboxymethyl)arginine
CML: Ne-(carboxymethyl)lysine
Col V: collagen V
COX-2: cyclooxygenase-2
CTGF: connective tissue growth factor
CVA16: Coxsackie virus A group 16 strain
Cyt c: cytochrome C
DCs: dendritic cells
E2: 17β-estradiol
E3: estrone
ECE: endothelin converting enzyme
ECM: extracellular matrix
ER^+^: estrogen receptor-positive
ERK1/2: extracellular-signal-related kinase 1/2
ET-1: endothelin-1
FN: fibronectin
GAP-43: growth-associated protein-43
GC–MS: gas chromatography–mass spectrometry
GPX: glutathione peroxidase
GSH: glutathione
GSP: glycated serum protein
GSSG: glutathione disulfide
HK-2: human renal proximal tubular epithelial cells
HO-1: heme oxygenase-1
HUVECs: human umbilical vein endothelial cells
ICAM-1: intercellular adhesion molecule-1
iNOS: inducible nitric oxide synthase
IFN: interferon
IL: interleukin
JNK: c-Jun N-terminal kinase
LPS: lipopolysaccharide
MAPK: mitogen-activated protein kinase
MCAO: middle cerebral artery occlusion
MCP-1: monocyte chemoattractant protein 1
MDA: malondialdehyde
MITF-M: microphthalmia-associated transcription factor-M
MMP: mitochondrial membrane potential
NF-kB: nuclear factor-kappa B
NFATc1: nuclear factor of activated T cells cytoplasmic 1
NGF: nerve growth factor
NO: nitric oxide
OSCAR: osteoclast-associated receptor
OVA: ovalbumin
*p*-eIF2α: *p*-eukaryotic initiation factor 2 alpha
PAECs: pulmonary artery endothelial cells
PEPCK: phosphoenolpyruvate carboxykinase
PERK: protein kinase R (PKR)-like endoplasmic reticulum kinase
PGE_2_: prostaglandin E_2_
PLB: phospholamban
p.o.: per os
RAGE: receptor of AGE
RANKL: receptor activator of nuclear factor kappa-Β ligand
ROS: reactive oxygen species
SERCA2a: sarcoplasmic reticulum Ca^2+^-ATPase 2a
SOD: superoxide dismutase
SPHK1: sphingosine kinase 1
SREBP-1&2: sterol regulatory element binding protein-1&2
STIM1: sensor protein stromal interaction molecule 1
STZ: streptozotocin
SYP: synaptophysin
TBARS: thiobarbituric acid-reactive substance
TC: triglyceride
TG: total cholesterol
TGF: transforming growth factor
TNF-α: tumor necrosis factor-α
TRAP: tartrate-resistant acid phosphatase
Trk A: tyrosine receptor kinase A
TRP-1&2: tyrosinase-related protein-1&2
VCAM-1: vascular cell adhesion molecule-1
VEGF: vascular endothelial growth factor
